# Genes and pathways underlying susceptibility to impaired lung function in the context of environmental tobacco smoke exposure

**DOI:** 10.1186/s12931-017-0625-7

**Published:** 2017-07-24

**Authors:** K. de Jong, J.M. Vonk, M. Imboden, L. Lahousse, A. Hofman, G.G. Brusselle, N.M. Probst-Hensch, D.S. Postma, H.M. Boezen

**Affiliations:** 1 Department of Epidemiology, University of Groningen, University Medical Center Groningen, Hanzeplein 1, 9700 RB Groningen, the Netherlands; 2 Groningen Research Institute for Asthma and COPD (GRIAC), University of Groningen, University Medical Center Groningen, Groningen, the Netherlands; 3 Swiss Tropical and Public Health Institute, Basel, Switzerland; 4 University of Basel, Basel, Switzerland; 5 Department of Epidemiology, Erasmus Medical Center, Rotterdam, the Netherlands; 6 Department of Respiratory Medicine, Ghent University Hospital, Ghent, Belgium; 7 Department of Internal Medicine, Erasmus Medical Center, Rotterdam, the Netherlands; 8 Department of Respiratory Medicine, Erasmus Medical Center, Rotterdam, the Netherlands; 9 Department of Pulmonary Diseases, University of Groningen, University Medical Center Groningen, Groningen, the Netherlands

**Keywords:** Environmental tobacco smoke pollution, Single nucleotide polymorphism, Forced expiratory volume

## Abstract

**Background:**

Studies aiming to assess genetic susceptibility for impaired lung function levels upon exposure to environmental tobacco smoke (ETS) have thus far focused on candidate-genes selected based on a-priori knowledge of potentially relevant biological pathways, such as *glutathione S-transferases* and *ADAM33*. By using a hypothesis-free approach, we aimed to identify novel susceptibility loci, and additionally explored biological pathways potentially underlying this susceptibility to impaired lung function in the context of ETS exposure.

**Methods:**

Genome-wide interactions of single nucleotide polymorphism (SNP) by ETS exposure (0 versus ≥1 h/day) in relation to the level of forced expiratory volume in one second (FEV_1_) were investigated in 10,817 subjects from the Dutch LifeLines cohort study, and verified in subjects from the Swiss SAPALDIA study (*n* = 1276) and the Dutch Rotterdam Study (*n* = 1156). SNP-by-ETS exposure *p*-values obtained from the identification analysis were used to perform a pathway analysis.

**Results:**

Fourty Five SNP-by-ETS exposure interactions with *p*-values <10^−4^ were identified in the LifeLines study, two being replicated with nominally significant *p*-values (<0.05) in at least one of the replication cohorts. Three pathways were enriched in the pathway-level analysis performed in the identification cohort LifeLines, i.E. the apoptosis, p38 MAPK and TNF pathways.

**Conclusion:**

This unique, first genome-wide gene-by-ETS interaction study on the level of FEV_1_ showed that pathways previously implicated in chronic obstructive pulmonary disease (COPD), a disease characterized by airflow obstruction, may also underlie susceptibility to impaired lung function in the context of ETS exposure.

**Electronic supplementary material:**

The online version of this article (doi:10.1186/s12931-017-0625-7) contains supplementary material, which is available to authorized users.

## Background

Detrimental effects of environmental tobacco smoke (ETS) exposure on the level of lung function have been shown in a number of studies. [1–3] Moreover, polymorphisms in several genes such as the glutathione S-transferases (*GSTs*), [4, 5] and *ADAM33,* [6] interact with ETS exposure, thereby negatively affecting the level and decline of lung function. In other words, ETS exposure has differential effects in subjects carrying mutant alleles compared to wild type alleles. Thus far, studies assessing interactions between genetic variants and ETS exposure were driven by a-priori knowledge of biological pathways, for example detoxification of noxious particles and gases by proteins such as the glutathione S-transferases.

In contrast, genome-wide interaction (GWI) studies are hypothesis-free and may yield novel loci in addition to those already known to be associated with impaired lung function directly (i.e. in genome-wide association studies without taking exposure into account). [7] Findings from such studies may provide novel insights in molecular pathways underlying disease pathogenesis, e.g. the development of chronic obstructive pulmonary disease (COPD) characterized by airflow obstruction. In this first genome-wide gene-by-ETS exposure study, we aimed to identify novel susceptibility loci, and additionally explored biological pathways potentially underlying this susceptibility to impaired forced expiratory volume in one second (FEV_1_) in the context of ETS exposure during adulthood.

## Methods

### Cohort and measurements

Subjects from the baseline investigation of the LifeLines cohort study (2006–2011), a multi-disciplinary prospective population-based cohort study examining health and health-related behaviour of persons living in the North East region of the Netherlands, were included as identification cohort. [8, 9] To verify our initial findings we included subjects from two independent general population-based cohorts, the Swiss SAPALDIA study, [10] and the Dutch Rotterdam Study. [11] More detailed description of the cohorts, spirometric measurements and genotyping can be found in the online supplement (Additional file 1: Methods).

### Exposure assessment

In the LifeLines and SAPALDIA studies ETS exposure was determined by the response to the question “how many hours per day are you exposed to other person’s tobacco smoke?”. In the Rotterdam study the question was similar, yet more specifically focused on exposure at home “at home, how many hours per day are you exposed to other person’s tobacco smoke?”. In all three cohorts, subjects were classified as ETS exposed if they reported at least 1 h/day (≥1 h/day) of exposure to other person’s tobacco smoke, and as non-exposed when self-reported ETS exposure was 0 h/day. Subjects that reported between 0 and 1 h/day of ETS exposure were excluded in order to have a clear exposure contrast.

### SNP-by-ETS exposure interactions

First, interactions of 227,981 genotyped SNPs with daily ETS exposure and their association with FEV_1_ were assessed in the identification cohort LifeLines. SNPs were tested in an additive genetic model including SNP, ETS exposure and the SNP-by-ETS exposure interaction. All models were adjusted for sex, age, height, ever smoking and pack years using linear regression models in the software package PLINK, version 1.07. [12] SNPs with *p*-values for the SNP-by-ETS interaction <10^−4^ were taken further for verification in the two independent cohorts, the SAPALDIA study and the Rotterdam Study (Additional file 1: Methods).

Finally SNP-by-ETS interaction effects from the three cohorts for the SNPs identified in the identification analysis were meta-analyzed using fixed effects models with effect estimates weighted by the inverse of the standard errors using software package METAL. [13] We selected SNP-by-ETS interactions with the same direction of interaction in all three cohorts and considered interactions with *p*-values < 0.05 in at least one of the two replication cohorts significantly replicated. SNP annotation was performed using HaploReg version 4.1. [14].

### Pathway analysis

All *p*-values for SNP-by-ETS interactions from the identification cohort LifeLines were used in the pathway analysis using the online improved gene set enrichment analysis tool i-GSEA-4-GWAS. [15] All log transformed *p*-values for SNPs 100 kb upstream and downstream of each gene were used to represent that specific gene. Each gene was represented by the lowest SNP *p*-value annotated to that gene. These SNP *p*-values were used to rank the genes, and the proportion of significant genes as a number of the total amount of genes (gene set) belonging to a pathway was calculated. Based on the rank, *p*-values were calculated for the association between the total gene set/pathway and the outcome. Additionally false discovery rate (FDR) corrected *p*-values were calculated. Gene sets/pathways with FDR corrected *p*-values < 0.25 are regarded as suggestively associated with the outcome, whereas FDR *p*-values < 0.05 are regarded as highly confident for an association with the outcome. [15].

## Results

### Descriptive statistics

In the LifeLines study, complete data on all covariates was available for 11,187 subjects. 370 subjects (3%) were excluded because they had self-reported ETS exposure between 0 and 1 h per day. Finally, the analysis included 10,817 subjects, of which 2473 subjects (23%) reported at least one hour of ETS exposure per day (Table 1). In the verification cohorts, complete data was available for 1276 subjects in the SAPALDIA study (23% being ETS exposed) and 1156 subjects in the Rotterdam study (19% being ETS exposed) (Table 1).

**Table 1:** Characteristics of the subjects included from the LifeLines, SAPALDIA and Rotterdam cohort studies

	LifeLines (identification)	SAPALDIA (replication)	Rotterdam Study 1 (replication)
N with complete data	10,817	1276	1156
ETS exposed (≥1 h/day), n (%)	2473 (23)	296 (23)	219 (19)
FEV_1_ (ml), mean (sd)	3401 (825)	3153 (864)	2199 (653)
FEV_1_% of predicted^a^, mean (sd)	102 (14)	96 (16)	104 (23)
FEV_1_/FVC (%), mean (sd)	76 (7)	74 (8)	75 (8)
FEV_1_/FVC < 70%, n (%)	1600 (15)	327 (26)	228 (20)
GOLD2+, n (%)	417 (4)	134 (11)	133 (12)
Use of medication for obstructive airway diseases^#^, n (%)	676 (6)	164 (18)^$^	106 (9)
Males, n (%)	4473 (41)	611 (48)	495 (43)
Age, median (min-max)	47 (18–88)	52 (29–72)	78 (72–95)
Ever smokers, n (%)	6266 (58)	703 (55)	771 (67)
Pack years in ever smokers, median (min-max)	10 (0–100)	12 (0–130)	18 (0–145)

*ETS* environmental tobacco smoke, *FEV*_*1*_ forced expiratory volume in one second, *FVC* forced vital capacity

^a^FEV_1_ as % of predicted following the reference equations of Quanjer et al. (1993) in LifeLines, [28] and GLI in Rotterdam study

GOLD2+ defined as FEV_1_/FVC < 70% and FEV_1_% of predicted <80%

^#^ATC code R03, ^$^ In SAPALDIA missing data for *n* = 377

### SNP-by-ETS exposure interactions

**Fig. 1:**
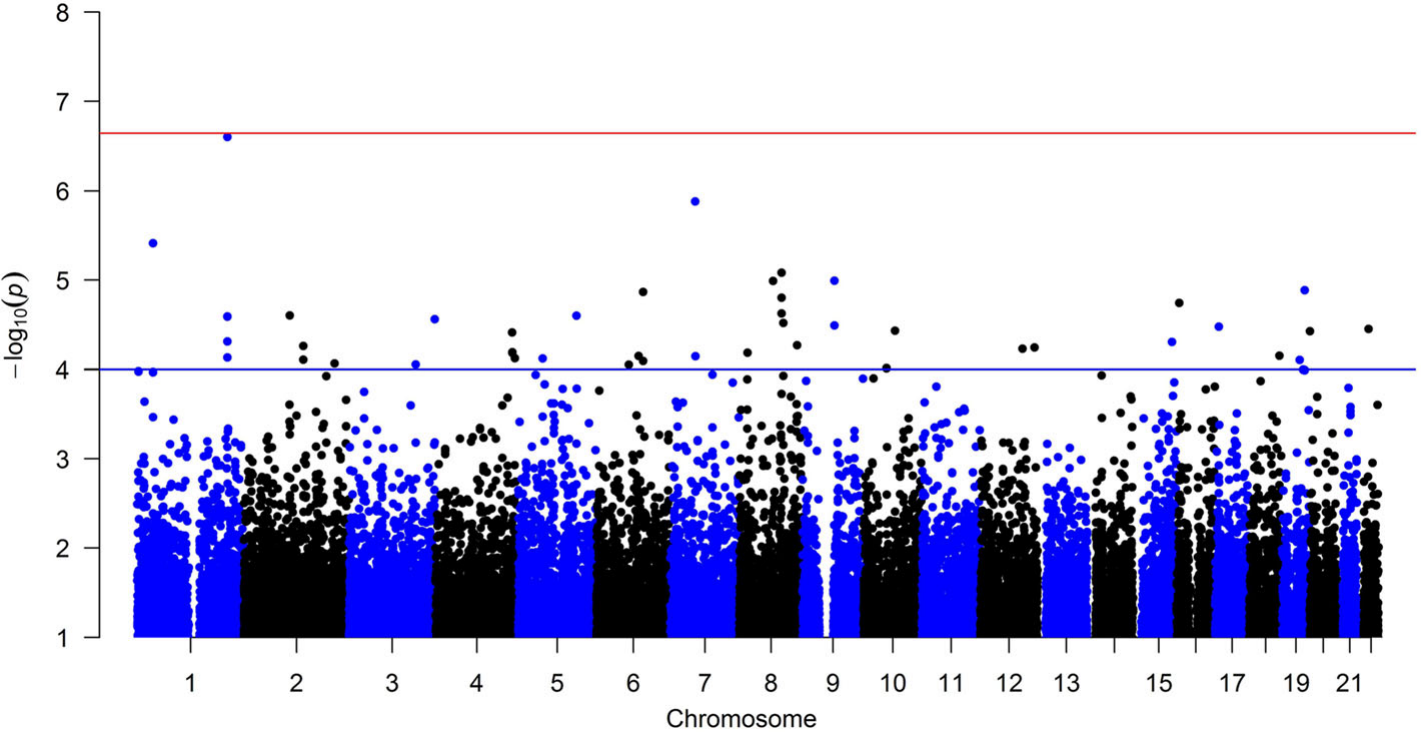
Manhattan plot showing SNP-by-ETS exposure interactions on the level of FEV_1_ in the identification cohort LifeLines. The blue line depicts the threshold for selection of SNPs (*p* = 10^−4^) and the red line the genome-wide significance level (*p* = 2.26*10^−7^)

*P*-values for each SNP-by-ETS exposure interaction in association with the level of FEV_1_ in the identification analysis are shown in a Manhattan plot (Fig. 1). A total of 45 SNPs had interaction *p*-values <10^−4^ in the identification cohort LifeLines (Additional file 2: Table S1) and were taken further for verification in the two independent cohorts. Of these 45 identified SNPs, rs4421160 was not available in the SAPALDIA study and two other SNPs, rs13282467 and rs743262, were not available in the Rotterdam Study.

Of the 45 identified SNP-by-ETS interactions, nine had the same direction of effect in all three cohorts. For all nine interactions there was little indication of heterogeneity between the cohorts (I^2^ for all interactions < 30) (Table 2). Two of these nine interactions were nominally significant (rs11950494 and rs2090789, *p* < 0.05) in one of the two verification cohorts. None of these nine interactions reached genome-wide significance in the meta-analysis of all three cohorts (i.e. Bonferroni-corrected threshold for 227,981 SNPs tested in the initial identification analysis = *p* < 2.26*10^−7^) (Table 2).

**Table 2:** Meta-analyzed SNP-by-ETS exposure interactions (additive for effect allele A1) with consistent direction of interaction effects in the three cohorts. Interactions effects for the independent cohorts LifeLines (identification), SAPALDIA (verification) and the Rotterdam Study (verification) are also shown

			Meta-analysis					LifeLines			SAPALDIA			Rotterdam Study		
SNP	A1	Gene (RefSeq)	B_int_	SE_int_	*P*-value	Direction	I^2^	B_int_	SE_int_	*P*-value	B_int_	SE_int_	*P*-value	B_int_	SE_int_	*P*-value
rs11950494	G	ACTBL2 (intronic)	−115	25	4.06E-06	---	0	−110	28	7.60E-05	−162	73	2.73E-02	−96	88	2.75E-01
rs2859741	T	GRIK3 (9.5 kb 5′)	59	13	6.78E-06	+++	0	69	15	3.86E-06	21	31	4.86E-01	38	51	4.61E-01
rs11153056	C	PDSS2 (intronic)	67	15	7.59E-06	+++	20	72	16	1.36E-05	69	44	1.15E-01	1	58	9.91E-01
rs7831729	C	SNX31 (15 kb 5′)	−76	17	1.12E-05	---	0	−80	19	3.03E-05	−43	49	3.88E-01	−79	62	1.99E-01
rs2084386	C	PAK2 (intronic)	90	21	1.47E-05	+++	7	95	23	2.74E-05	28	71	6.90E-01	101	74	1.74E-01
rs9386622	T	PDSS2 (intronic)	63	15	1.67E-05	+++	25	65	16	8.08E-05	82	43	5.55E-02	22	51	6.74E-01
rs2090789	A	LOC100128993 (intronic)	−53	13	3.95E-05	---	0	−60	15	6.54E-05	−9	31	7.71E-01	−99	50	4.77E-02
rs7526579	C	KCNH1 (intronic)	−67	16	4.37E-05	---	29	−75	18	4.88E-05	−18	44	6.79E-01	−72	60	2.32E-01
rs7030493	T	TMEM2 (intronic)	−101	25	4.99E-05	---	0	−112	27	3.24E-05	−42	86	6.25E-01	−30	98	7.59E-01

### Pathway analysis

Of all SNP-by-ETS interactions included in the identification analysis in the LifeLines study, *p*-values of 165,298 interactions were mapped to 15,243 genes and 231 gene sets/pathways. Pathway analysis showed one significant (FDR *p*-value < 0.05) and two suggestively enriched pathways (FDR *p*-value < 0.25) (Table 3). The most significant, i.e. the apoptosis pathway, includes 71 genes of which 54 were present in the identification analysis and 23 were significantly associated with the outcome (Table 4). The two suggestively associated pathways were the p38 MAPK pathway (Table 5) and tumor necrosis factor (TNF) pathway (Table 6), with 16 genes and 9 genes from the SNP-by-ETS exposure interaction analysis that were significantly associated with the outcome, respectively.

**Table 3:** Significantly (FDR *p*-value <0.05) and suggestively (FDR *p*-value <0.25) enriched pathways based on genome-wide interaction SNP-by-ETS exposure *p*-values within 100 kb of the gene using i-GSEA-4-GWAS [15]

Pathway/Gene set name	Description	FDR corrected *p*-value	Significant genes/ Selected genes/All genes
Apoptosis	Apoptosis is a distinct form of cell death that is functionally and morphologically different from necrosis.	0.003	22/54/71
P38 MAPK pathway	The Rho family GTPases activate the p38 MAPKs under environmental stress or in the presence of pro-inflammatory cytokines.	0.063	16/33/40
Tumor Necrosis Factor pathway	Tumor necrosis factor is a pro-inflammatory cytokine that activates NF-kB and c-Jun.	0.081	9/22/29

**Table 4:** Genes significantly enriched in the apoptosis pathway. Effect estimates (B_int_ in mL FEV_1_) and *p*-values from the SNP-by-ETS exposure interaction analysis in LifeLines are given

Significant genes in the Apoptosis pathway				
Pathway analysis			SNP-by-ETS exposure interaction	
Gene Name	SNP	-log(*P*-value)	B_int_ FEV_1_ (ml)	*P*-value
APAF1	rs12581724	4.23	−111	5.88E-05
MYC	rs7829529	3.00	109	1.00E-03
IRF2	rs3775574	2.74	−47	1.81E-03
BNIP3L	rs3808578	2.63	−70	2.35E-03
CASP9	rs4645983	2.52	−52	3.00E-03
BCL2L11	rs1877330	2.47	85	3.40E-03
JUN	rs2716129	2.45	−43	3.52E-03
MAP2K4	rs2013868	2.11	−51	7.73E-03
TNFRSF10B	rs11784599	1.91	−48	1.24E-02
MAPK10	rs7688651	1.86	38	1.39E-02
FAS	rs1926189	1.75	54	1.76E-02
GZMB	rs1957519	1.75	−62	1.79E-02
NFKBIE	rs513688	1.72	37	1.91E-02
TNFRSF1B	rs235214	1.67	50	2.14E-02
BCL2L1	rs6060870	1.64	42	2.29E-02
FADD	rs10751209	1.57	−33	2.71E-02
BAK1	rs210138	1.55	42	2.80E-02
BCL2	rs1801018	1.50	32	3.17E-02
CASP8	rs3769823	1.47	35	3.40E-02
HRK	rs4767462	1.46	−63	3.43E-02
TNFRSF21	rs9463313	1.46	37	3.46E-02
NFKB1	rs2085548	1.38	33	4.17E-02

**Table 5:** Genes significantly enriched in the p38 MAPK pathway. Effect estimates (B_int_ in mL FEV_1_) and *p*-values from the SNP-by-ETS exposure interaction analysis are given

Significant genes in the p38 MAPK pathway				
Pathway analysis			SNP-by-ETS exposure interaction	
Gene Name	SNP	-log(*P*-value)	B_int_ FEV_1_ (ml)	*P*-value
MYC	rs7829529	4.23	109	1.00E-03
TGFBR1	rs12686783	3.00	71	1.39E-03
MAP2K6	rs2716227	2.74	−45	2.36E-03
MAP2K4	rs2013868	2.63	−51	7.73E-03
CREB1	rs722761	2.52	−38	1.38E-02
MAPK14	rs851023	2.47	52	1.47E-02
MEF2A	rs7164257	2.45	−55	1.80E-02
MAP3K9	rs731571	2.11	42	2.01E-02
TGFB2	rs2798631	1.91	35	2.05E-02
HSPB1	rs2908201	1.86	−39	2.22E-02
MAX	rs2763887	1.75	−67	3.32E-02
MAP3K7	rs205349	1.75	50	3.38E-02
MAP3K5	rs2237268	1.72	32	3.41E-02
PLA2G4A	rs10489409	1.67	−53	3.81E-02
MAPKAPK2	rs11119447	1.64	−32	3.87E-02
HMGN1	rs2836992	1.57	−31	3.93E-02

**Table 6:** Genes significantly enriched in the Tumor Necrosis Factor pathway. Effect estimates (B_int_ in mL FEV_1_) and *p*-values from the SNP-by-ETS exposure interaction analysis in LifeLines are given

Significant genes in the Tumor Necrosis Factor pathway				
Pathway analysis			SNP-by-ETS exposure interaction	
Gene Name	SNP	-log(*P*-value)	B_int_ FEV_1_ (ml)	*P*-value
JUN	rs2716129	2.45	−43	3.52E-03
MAP2K4	rs2013868	2.11	−51	7.73E-03
TNFAIP3	rs11970361	1.94	−95	1.14E-02
NFKBIE	rs513688	1.72	37	1.91E-02
TNFRSF1B	rs235214	1.67	50	2.14E-02
FADD	rs10751209	1.57	−33	2.71E-02
MAP3K7	rs205349	1.47	50	3.38E-02
CASP8	rs3769823	1.47	35	3.40E-02
NFKB1	rs2085548	1.38	33	4.17E-02

## Discussion

The current study is the first to explore SNP-by-ETS exposure interactions on the level of FEV_1_ during adulthood in a hypothesis-free genome-wide manner. We extended our findings to pathway level analysis and showed that several pathways, i.e. the apoptosis, p38 MAPK and TNF pathways, may be underlying susceptibility to impaired FEV_1_ in the context of ETS exposure.

The SNP with the most significant interaction in the identification cohort was located in the gene coding for *KCNH1,* also known as ether-à-go-go *(EAG1)*. KCNH1 is a voltage-gated potassium channel that is highly expressed on mast cells and macrophages in germinal centers of reactive lymph nodes, [16] which may indicate its involvement in immune responses. Moreover, both mRNA and protein expression of *KCNH1* were up-regulated during epithelial-to-mesenchymal transition (EMT) of human lung tumor cells induced by TGFβ1. [17] Increased expression of EMT markers has been observed in the airways of smokers, especially those with COPD. [18] Although one SNP in *KCNH1* (rs7526579) had the same direction of interaction effect in all three cohorts, it did not reach genome-wide significance after meta-analysis and did not reach nominal significance in at least one of the verification cohorts. Findings, therefore, remain speculative.

Two SNPs-by-ETS interactions identified in the LifeLines study had nominally significant *p*-values in at least one of the verification cohorts, i.e. rs11950494 in SAPALDIA and rs2090789 in the Rotterdam Study. SNP rs11950494 is located in the gene actin beta-like 2 (*ACTBL2*) and rs2090789 is located in a predicted non-coding RNA *LOC100128993*. Both genes are expressed in lung tissue (genecards.org), however, at current little is known about their biological function in general or relevance to lung function specifically.

In the current study, we used a large and well documented homogeneous cohort of a general population, i.e. the LifeLines study, to assess SNP-by-ETS exposure interactions. We used a liberal *p*-value threshold (*p* < 10^−4^) in the identification analysis and attempted to verify the SNP-by-ETS exposure interactions in two independent cohorts, the SAPALDIA and Rotterdam studies. Only 2 SNP-by-ETS exposure interactions were replicated with a nominal *p*-value and with the same direction of effect, which is less than expected based on chance only (i.e. 5% of 45 SNPs = 2.25). Moreover, none of the SNPs reached the Bonferroni-corrected threshold for genome-wide significance (*p*-value = 2.19*10^−7^). Therefore interpretation of the results remains difficult and the implications of the outcomes uncertain. The replication cohorts were relatively small, which may have limited the power to significantly replicate our findings. Another reason for not finding significant interaction effects may be the rather crude assessment of ETS exposure. In general, measuring ETS exposure during adulthood is difficult, especially when using self-reports. Thus far, no GWI studies on ETS exposure during adulthood have been published, suggesting that either no studies have been performed, or that publication bias exists due to null findings. There were slight differences in characteristics between the cohorts, i.e. enrichment with asthmatics in SAPALDIA (40% asthmatics) and the older mean age of subjects in the Rotterdam Study. However, sensitivity analysis in the identification cohort suggested that effects estimates for the two marginally replicated SNPs did not change when only non-asthmatics were included. Moreover, SNP-by-ETS interaction effects rather get more than less pronounced in older (≥50 years) compared to younger subjects (<50 years) (data not shown). Interestingly, a final sensitivity analysis showed that associations of these two SNP-by-ETS interactions with FEV_1_, did only remain in subjects without airway obstruction (FEV_1_/FVC ≥ 70%) (data not shown), suggesting that genetic susceptibility to effects of ETS is less important when already having airway obstruction.

In addition to single SNP analysis we performed a pathway analysis based on interaction *p*-values in the LifeLines study. Compared to single SNP analysis, pathway analysis may have increased power to detect genetic associations of the phenotype with a gene set/pathway. [19] Three pathways were significantly or suggestively enriched, i.e. the apoptosis, p38 MAPK and TNF pathways. Interestingly all three pathways may mutually interact and have been previously implicated in the pathogenesis of COPD, a disease caused by an abnormal inflammatory response to noxious particles and gases leading to airflow obstruction.

Apoptosis is a programmed form of cell death. Previous investigations within the SAPALDIA study have found suggestive evidence that genetic variation in the apoptosis pathway modifies the effect of pack years smoked on the decline of FEV_1_. [20] An imbalance between apoptosis and proliferation of alveolar epithelial and endothelial cells has been observed in the lungs of patients with COPD. [21] Apoptosis is regulated by various pathways. One of the pathways is a response to extracellular signals by binding of members of the tumor necrosis family, such as TNF-alpha with death receptor TNF-receptor 1. [21] For example, cigarette smoke exposure was shown to increase TNF-alpha expression. [22] This interaction between the different pathways was also reflected by the substantial overlap in genes enriched in the TNF-alpha (Table 6) and apoptosis pathways (Table 4) in the pathway analysis. Another pro-apoptotic pathway responds to physical and chemical stressors via the release of cytochrome C by mitochondria. Subsequent formation of an apoptosome activates several caspases which eventually initiate apoptosis. Interestingly, we identified an intronic SNP in *APAF1* that interacted with ETS exposure (*p*-value = 5.88*10^−5^) in the identification cohort LifeLines, the expressed protein of this gene is part of this apoptosome initiating apoptosis (Additional file 1: Tables S1 and Table 4). However, this SNP-by-ETS exposure interaction was not replicated in the SAPALDIA or Rotterdam study.

The TNF pathway was suggestively enriched in the pathway analysis. TNF-alpha is a cytokine playing an important role in inflammation through its activation of several downstream signaling cascades, amongst others the p38 MAPK pathway. Levels of TNF-alpha have been shown to be increased in sputum of COPD patients compared to both non-smoking and smoking controls, and in response to air pollution exposure. [23, 24] The second suggestively enriched pathway was the p38 mitogen activated protein kinase (MAPK) pathway, this pathway has also been implicated in the development and/or maintenance of a number of chronic airway inflammatory diseases such as COPD. [25] The p38 MAPK pathway is activated by various environmental stressors, growth factors and cytokines and in turn regulates the expression of inflammatory cytokines such as TNF-alpha and may initiate apoptosis. [26] Increased activation of p38 MAPK was seen in alveolar walls and alveolar macrophages of COPD patients compared to non-smoking and smoking controls. [27].

## Conclusion

The current study is the first to explore gene-by-ETS interactions related to the level of FEV_1_ in a hypothesis-free genome-wide manner. Findings from the single SNP analysis remain uncertain, yet our pathway analysis suggests that pathways previously implicated in COPD pathogenesis may, as well, underlie susceptibility to impaired lung function in the context of ETS exposure.

## Additional files

Additional file 1: Supplementary Methods. (DOCX 20 kb)

Additional file 2: Table S1. Interactions between SNPs (additive effect for minor allele A1) and ETS exposure on the level of FEV_1_ (ml) with *p*-values <10^−4^ in the identification analysis (LifeLines). Linear regression models were adjusted for sex, age, height, ever smoking and pack years smoked. MAF is given for the effect allele (A1). (DOCX 26 kb)

## Abbreviations

COPD: Chronic obstructive pulmonary disease
ETS: Environmental tobacco smoke
FDR: False discovery rate
FEV_1_: Forced expiratory volume in one second
GSTs: Glutathione S-transferases
GWI: Genome-wide interaction
SNP: Single nucleotide polymorphism
TNF: Tumor necrosis factor
MAPK: P38 mitogen activated protein kinase
FVC: Forced vital capacity
