# Supplementary material for: Genes and pathways underlying susceptibility to impaired lung function in the context of environmental tobacco smoke exposure
Source: Respir Res. 2017 Jul 24;18:142. doi: 10.1186/s12931-017-0625-7 (PMC5525356; doi:10.1186/s12931-017-0625-7)
Supplement: Supplementary file 1 — Supplementary Methods. (DOCX 20 kb) [file 12931_2017_625_MOESM1_ESM.docx]

**SUPPLEMENTARY METHODS**

**Genes and pathways underlying susceptibility to impaired lung function in the context of environmental tobacco smoke exposure**

K. de Jong^1,2^, J.M. Vonk^1,2^, M. Imboden^3,4^, L. Lahousse^5,6^, A. Hofman^5,7^, G.G Brusselle^5,6,8^, N.M. Probst-Hensch^3,4^, D.S. Postma^9,2^, H.M. Boezen^1,2^.

^1^ University of Groningen, University Medical Center Groningen, Department of Epidemiology, Groningen, the Netherlands.

^2^ University of Groningen, University Medical Center Groningen, Groningen Research Institute for Asthma and COPD (GRIAC), Groningen, the Netherlands.

^3^ Swiss Tropical and Public Health Institute, Basel, Switzerland.

^4^ University of Basel, Basel, Switzerland.

^5^ Erasmus Medical Center, Department of Epidemiology, Rotterdam, the Netherlands.

^6^ Ghent University Hospital, Department of Respiratory Medicine, Ghent, Belgium.

^7^ Erasmus Medical Center, Department of Internal Medicine, Rotterdam, the Netherlands.

^8^ Erasmus Medical Center, Department of Respiratory Medicine, Rotterdam, the Netherlands.

^9^ University of Groningen, University Medical Center Groningen, Department of Pulmonary Diseases, Groningen, the Netherlands.

**Corresponding author:**

H.M. Boezen, University of Groningen, University Medical Center Groningen, Department of Epidemiology, Hanzeplein 1, 9700 RB Groningen, the Netherlands.

E-mail address: h.m.boezen@umcg.nl

Phone number: +31 50 3610899

**DESCRIPTION OF THE COHORTS**

**LifeLines cohort study**

The LifeLines cohort study started in 2006, including subjects from the three Northern provinces of the Netherlands [1,2]. At baseline all participants had a blood sample taken, filled in a standardized questionnaire and were subject to a medical examination including pre-bronchodilator spirometry following American Thoracic Society criteria. The LifeLines cohort study was approved by the Medical Ethics Committee of the University Medical Center Groningen, Groningen, The Netherlands. All subjects gave written informed consent. Blood from a subset of subjects, all with baseline visits between December 2006 and February 2011, was used for genotyping. Genotyping was performed using IlluminaCytoSNP-12 arrays. SNPs (n = 227,981) that fulfilled the quality control criteria were included: genotype call-rate ≥ 95%, minor allele frequency ≥ 1%, and Hardy-Weinberg equilibrium cut-off p-value ≥ 10^-4^. Samples with call rates below 95% were excluded. First degree relatives and non-Caucasian samples, based on self-report, outlier (IBS) and principal component analysis, were excluded.

**SAPALDIA study**

The phenotype data from the SAPALDIA study was derived from the first follow-up survey collected in 2002 [3]. The study was enriched with asthmatics (40%). Participants provided informed consent for participation in the health interviews, physical examinations, blood marker and genetic assays. Ethical clearance for the SAPALDIA study was obtained from the Swiss Academy of Medical Sciences, the National Ethics Committee for Clinical research (UREK, Project Approval Number 123/00) and the Ethics Committees of the eight participating communities including Basel, Wald, Davos, Lugano, Montana, Payerne, Aarau and Geneva.

Pre-bronchodilator spirometry was performed according to a standardized protocol equivalent to that of the European Community Respiratory Health Survey (ECRHS), using a Sensormedics model 2200 (Yorba Linda, California, USA) and following American Thoracic Society criteria. Blood samples were used to genotype 567,589 SNPs using the Illumina 610K quad array. Consequently this sample was imputed using MACH v1.00 software 13 and the HapMap2 Release 22 CEU reference sample. To account for population stratification, ancestry-informative principal components were inferred using software package EIGENSTRAT2.0 using HapMap data (CEU, YRI, JPT and CHB) and additional European reference samples. Non-European and related samples were excluded.

SNP-by-ETS exposure interactions were assessed with linear regression models adjusted for adjusted for sex, age, height, ever smoking and pack years, study area and principal components ev3 and ev4 using the software packages STATA MP12 and PLINK version 1.07 [4].

**Rotterdam Study I**

The Rotterdam Study is a prospective population-based study investigating chronic diseases in elderly living in the Ommoord district in the city of Rotterdam in the Netherlands [5]. For the current analysis subjects of the Rotterdam Study I (RS1) were included, these were subjects aged 55 years and older which visited the research center for the first time between July 1989 and September 1993. Lung function data for this analysis was derived from pre-bronchodilator spirometry which was performed between March 2009 and January 2011 using a Master Screen® PFT Pro (CareFusion, San Diego, CA) by trained paramedical personnel according to the ATS/ERS guidelines. All subjects provided written informed consent and the study was approved by the medical ethics committee of Erasmus University. A total of 6,318 subjects were genotyped in RS I (Illumina 550(+duo)/Illumina 610 quad). Exclusions included a call rate < 98%, Hardy-Weinberg p-value < 10^-6^ and MAF < 0.01%. A total of 6,291 samples passed genotyping quality control.

SNP-by-ETS exposure interactions were assessed in linear regression models adjusted for sex, age, height, ever smoking and pack years using the ProbABEL program according to an additive model using the 1000 genomes 1KG_phaseIv3 imputation panel [6]. All subjects in the final analysis (n = 1,156) were of Caucasian ancestry.

**References**

1. Stolk R, Rosmalen JGM, Postma D, de Boer R, Navis G, Slaets JPJ, et al. Universal risk factors for multifactorial diseases: LifeLines: a three-generation population-based study. Eur J Epidemiol. 2008;23: 67-74.

2. Scholtens S, Smidt N, Swertz MA, Bakker SJ, Dotinga A, Vonk JM, et al. Cohort Profile: LifeLines, a three-generation cohort study and biobank. Int J Epidemiol. 2015;44: 1172-1180.

3. Leuenberger P, Schwartz J, Ackermann Liebrich U, Blaser K, Bolognini G, Bongard JP, et al. Passive smoking exposure in adults and chronic respiratory symptoms (SAPALDIA Study). Swiss Study on Air Pollution and Lung Diseases in Adults, SAPALDIA Team. . 1994;150: 1222-1228.

4. Purcell S, Neale B, Todd-Brown K, Thomas L, Ferreira MA, Bender D, et al. PLINK: a tool set for whole-genome association and population-based linkage analyses. Am J Hum Genet. 2007;81: 559-575.

5. Hofman A, Brusselle GG, Darwish Murad S, van Duijn CM, Franco OH, Goedegebure A, et al. The Rotterdam Study: 2016 objectives and design update. Eur J Epidemiol. 2015;30: 661-708.

6. Kreiner-Moller E, Medina-Gomez C, Uitterlinden AG, Rivadeneira F, Estrada K. Improving accuracy of rare variant imputation with a two-step imputation approach. Eur J Hum Genet. 2015;23: 395-400.
