# Supplementary material for: Genes and pathways underlying susceptibility to impaired lung function in the context of environmental tobacco smoke exposure
Source: Respir Res. 2017 Jul 24;18:142. doi: 10.1186/s12931-017-0625-7 (PMC5525356; doi:10.1186/s12931-017-0625-7)
Supplement: Supplementary file 2 — Interactions between SNPs (additive effect for minor allele A1) and ETS exposure on the level of FEV1 (ml) with p-values <10−4 in the identification analysis (LifeLines). Linear regression models were adjusted for sex, age, height, ever smoking and pack years smoked. MAF is given for the effect allele (A1). (DOCX 26 kb) [file 12931_2017_625_MOESM2_ESM.docx]

**SUPPLEMENTARY TABLE S1**

**Genes and pathways underlying susceptibility to impaired lung function in the context of environmental tobacco smoke exposure**

K. de Jong^1,2^, J.M. Vonk^1,2^, M. Imboden^3,4^, L. Lahousse^5,6^, A. Hofman^5,7^, G.G Brusselle^5,6,8^, N.M. Probst-Hensch^3,4^, D.S. Postma^9,2^, H.M. Boezen^1,2^.

^1^ University of Groningen, University Medical Center Groningen, Department of Epidemiology, Groningen, the Netherlands.

^2^ University of Groningen, University Medical Center Groningen, Groningen Research Institute for Asthma and COPD (GRIAC), Groningen, the Netherlands.

^3^ Swiss Tropical and Public Health Institute, Basel, Switzerland.

^4^ University of Basel, Basel, Switzerland.

^5^ Erasmus Medical Center, Department of Epidemiology, Rotterdam, the Netherlands.

^6^ Ghent University Hospital, Department of Respiratory Medicine, Ghent, Belgium.

^7^ Erasmus Medical Center, Department of Internal Medicine, Rotterdam, the Netherlands.

^8^ Erasmus Medical Center, Department of Respiratory Medicine, Rotterdam, the Netherlands.

^9^ University of Groningen, University Medical Center Groningen, Department of Pulmonary Diseases, Groningen, the Netherlands.

**Corresponding author:**

H.M. Boezen, University of Groningen, University Medical Center Groningen, Department of Epidemiology, Hanzeplein 1, 9700 RB Groningen, the Netherlands.

E-mail address: h.m.boezen@umcg.nl

Phone number: +31 50 3610899

**Table S1. Interactions between SNPs (additive effect for minor allele A1) and ETS exposure on the level of FEV_1_ (ml) with p-values < 10^-4^ in the identification analysis (LifeLines). Linear regression models were adjusted for sex, age, height, ever smoking and pack years smoked. MAF is given for the effect allele (A1).**

|  |  |  |  | **95% CI** | |  |  |  | **Functional annotation** | **LD** |
| --- | --- | --- | --- | --- | --- | --- | --- | --- | --- | --- |
| **chr** | **SNP** | **A1** | **B_int_** | **Lower** | **Upper** | **P-value** | **MAF** | **Gene** |  | **R^2^** |
| 1 | rs2859741 | T | 69 | 40 | 98 | 3.86E-06 | 0.45 | 9.5kb 5' of GRIK3 |  |  |
| 1 | rs7526579 | C | -75 | -111 | -39 | 4.88E-05 | 0.20 | KCNH1 | intronic |  |
| 1 | rs1846946 | T | 63 | 32 | 94 | 7.41E-05 | 0.33 | KCNH1 | intronic |  |
| 1 | rs924568 | G | 79 | 49 | 109 | 2.49E-07 | 0.38 | KCNH1 | intronic | LD with rs924568 > |
| 1 | rs4951491 | C | 67 | 36 | 98 | 2.58E-05 | 0.32 | KCNH1 | intronic | rs924568 |
| 1 | rs6540647 | G | 63 | 34 | 92 | 2.57E-05 | 0.44 | KCNH1 | intronic | > 0.76 |
| 2 | rs17031275 | A | 120 | 64 | 176 | 2.49E-05 | 0.08 | 31kb 3' of LOC285000 |  |  |
| 2 | rs4954603 | G | 93 | 48 | 138 | 5.51E-05 | 0.12 | 185kb 5' of CXCR4 |  | 0.54 |
| 2 | rs7570134 | G | 75 | 38 | 112 | 7.84E-05 | 0.20 | 210kb 5' of CXCR4 |  |  |
| 2 | rs10497902 | C | 78 | 39 | 117 | 8.62E-05 | 0.17 | 77kb 3' of PTH2R |  |  |
| 3 | rs528581 | C | 60 | 30 | 90 | 8.88E-05 | 0.37 | 75kb 5' of RAP2B |  |  |
| 3 | rs2084386 | C | 95 | 51 | 140 | 2.74E-05 | 0.12 | PAK2 | intronic |  |
| 4 | rs17062990 | G | -102 | -150 | -53 | 3.87E-05 | 0.10 | 118kb 3' of SPCS3 |  | LD with rs17062990 > 0.74 |
| 4 | rs4146433 | T | -89 | -133 | -45 | 6.44E-05 | 0.13 | 147kb 3' of SPCS3 |  |  |
| 4 | rs11133161 | A | -92 | -138 | -47 | 6.58E-05 | 0.11 | 138kb 3' of VEGFC |  |  |
| 4 | rs4861505 | G | -68 | -102 | -34 | 7.53E-05 | 0.25 | ODZ3 | intronic |  |
| 5 | rs11950494 | G | -110 | -164 | -55 | 7.60E-05 | 0.08 | ACTBL2 | 3'-UTR |  |
| 5 | rs1393082 | A | 68 | 36 | 99 | 2.51E-05 | 0.30 | H2AFY | intronic |  |
| 6 | rs4421160 | T | -90 | -135 | -45 | 8.89E-05 | 0.11 | 144kb 3' of CD109 |  |  |
| 6 | rs982124 | A | -294 | -439 | -149 | 7.12E-05 | 0.01 | KLHL32 | intronic |  |
| 6 | rs9386622 | T | 65 | 33 | 97 | 8.08E-05 | 0.29 | PDSS2 | intronic | 0.60 |
| 6 | rs11153056 | C | 72 | 39 | 104 | 1.36E-05 | 0.29 | PDSS2 | intronic |  |
| 7 | rs1533956 | G | 74 | 44 | 104 | 1.31E-06 | 0.34 | 12kb 5' of MIR3147 |  | 0.74 |
| 7 | rs11135646 | T | 64 | 32 | 96 | 7.15E-05 | 0.28 | 18kb 5' of ZNF716 |  |  |
| 8 | rs2090789 | A | -60 | -89 | -30 | 6.54E-05 | 0.46 | LOC100128993 | intronic |  |
| 8 | rs2733727 | A | 144 | 80 | 208 | 1.02E-05 | 0.05 | ZFHX4 | intronic |  |
| 8 | rs16894633 | C | 118 | 66 | 171 | 8.31E-06 | 0.08 | 6.2kb 5' of SDC2 |  | 0.92 |
| 8 | rs16894649 | A | 111 | 59 | 162 | 2.37E-05 | 0.08 | SDC2 | intronic |  |
| 8 | rs12056723 | G | -131 | -191 | -72 | 1.58E-05 | 0.06 | SDC2 | intronic |  |
| 8 | rs7831729 | C | -80 | -118 | -43 | 3.03E-05 | 0.18 | 15kb 5' of SNX31 |  |  |
| 8 | rs13282467 | A | -195 | -290 | -100 | 5.37E-05 | 0.02 | LRRC6 | intronic |  |
| 9 | rs7030493 | T | -112 | -165 | -59 | 3.24E-05 | 0.08 | TMEM2 | intronic | 0.93 |
| 9 | rs1552708 | G | -117 | -168 | -65 | 1.02E-05 | 0.08 | TMEM2 | intronic |  |
| 10 | rs2174257 | G | -59 | -89 | -29 | 9.69E-05 | 0.39 | PRKG1 | intronic |  |
| 10 | rs2593163 | G | 103 | 54 | 152 | 3.71E-05 | 0.09 | PSAP | intronic |  |
| 12 | rs12581724 | C | -111 | -165 | -57 | 5.88E-05 | 0.08 | APAF1 | intronic |  |
| 12 | rs225574 | T | -78 | -116 | -40 | 5.70E-05 | 0.18 | LOC400084 | intronic |  |
| 15 | rs6496799 | G | -80 | -118 | -41 | 4.95E-05 | 0.18 | 124kb 3' of SV2B |  |  |
| 16 | rs8052564 | A | 120 | 65 | 175 | 1.81E-05 | 0.08 | RBFOX1 | intronic |  |
| 17 | rs8067644 | T | -65 | -95 | -34 | 3.34E-05 | 0.35 | PIK3R6 | intronic |  |
| 18 | rs7233554 | C | -108 | -161 | -55 | 7.08E-05 | 0.09 | 20kb 3' of CYB5A |  |  |
| 19 | rs7976 | T | 71 | 36 | 106 | 7.88E-05 | 0.22 | KRTDAP | 3'-UTR |  |
| 19 | rs311384 | G | 70 | 39 | 102 | 1.31E-05 | 0.31 | ARHGAP35 | intronic |  |
| 20 | rs753320 | A | -89 | -132 | -47 | 3.76E-05 | 0.13 | 27kb 5' of SCRT2 |  |  |
| 22 | rs743262 | A | 274 | 144 | 404 | 3.53E-05 | 0.01 | 206kb 3' of MN1 |  |  |
